# Both Humoral and Cellular Immune Responses to SARS-CoV-2 Are Essential to Prevent Infection: a Prospective Study in a Working Vaccinated Population from Southern France

**DOI:** 10.1007/s10875-023-01558-9

**Published:** 2023-08-22

**Authors:** Daisy Graça, Vesna Brglez, Jonathan Allouche, Kévin Zorzi, Céline Fernandez, Maxime Teisseyre, Marion Cremoni, Sylvia Benzaken, Christian Pradier, Barbara Seitz-Polski

**Affiliations:** 1 Centre Hospitalier Universitaire de Nice, Laboratoire d’immunologie, Nice, France; 2 Université Côte d’Azur – Centre Hospitalier Universitaire de Nice, UR2CA, Nice, France; 3 Centre Hospitalier Universitaire de Nice, Département de Santé Publique, Nice, France

**Keywords:** COVID-19, vaccination, B cell response, T cell response, hybrid immunity, SARS-Cov-2

## Abstract

COVID-19 vaccines have significantly decreased the number of severe cases of the disease, but the virus circulation remains important, and questions about the need of new vaccination campaigns remain unanswered. The individual’s protection against SARS-CoV-2 infection is most commonly measured by the level and the neutralizing capacity of antibodies produced against SARS-CoV-2. T cell response is a major contributor in viral infection, and several studies have shown that cellular T cell response is crucial in fighting off SARS-CoV-2 infection. Actually, no threshold of protective immune response against SARS-CoV2 infection has been identified. To better understand SARS-CoV-2-mediated immunity, we assessed both B cell (measuring anti-Spike IgG titer and neutralization capacity) and T cell (measuring IFNγ release assay after specific SARS-CoV2 stimulation) responses to SARS-CoV-2 vaccination with or without virus encounter in a cohort of 367 working volunteers. Vaccinated individuals who had previously been infected had a stronger and more lasting immunity in comparison to vaccinated individuals naive to infection whose immunity started to decline 3 months after vaccination. IFNγ release ≥ 0.285 IU/mL and anti-Spike IgG antibodies ≥ 244 BAU/mL were associated with a sufficient immune response following vaccination preventing future infections. Individuals with comorbidities had a lower chance of reaching the protective thresholds of T cell and B cell responses as identified in multivariate analysis. A combined B cell and T cell analysis of immune responses to determine protective thresholds after SARS-CoV-2 vaccination will allow us to identify individuals in need of a booster vaccine dose, particularly in comorbid subjects.

## Introduction

Coronavirus disease 2019 (COVID-19) caused by severe acute respiratory syndrome coronavirus 2 (SARS-CoV-2) has emerged in December 2019 and has since spread across the world, causing a worldwide pandemic. In an attempt to limit the spread of the virus, large-scale vaccination has since become the main public health measure in most countries. Nevertheless, the virus continues to circulate, and new variants keep emerging. It is therefore of outmost importance to understand how best to measure the efficacy of vaccination- or infection-procured immunity of the population [1, 2].

Only very recently the data has begun to emerge on the comparative efficiency of vaccination or natural infection or a combination of both against future breakthrough infections [3]. This is especially important given the rapid emergence and spread of new variants, such as Omicron, which pose a new threat to otherwise seemingly protected nearly fully vaccinated population. Indeed, it seems that the protective effect of either previous infection or vaccination is diminished for Delta and Omicron variants in comparison to the Wuhan variant or other early variants of concern [4–6]. Several factors, in addition to well-known and often described clinical and demographic parameters, may be at play when determining the risk of reinfection, such as the type of vaccine or the variant in the case of previous natural infection, as well as the time elapsed since the last vaccination/infection event [2, 3, 7].

The individual’s protection against SARS-CoV-2 infection is most commonly measured by the level and the neutralizing capacity of antibodies produced against SARS-CoV-2 either as a result of a previous infection or vaccination [1]. However, the measurement of humoral B cell response gives only a fraction of information about an individual’s capability to fight off SARS-CoV-2 infection. In a viral infection, T cell response is a major contributor and several recent studies have shown that cellular T cell response, often neglected in large-scale cohort studies, is crucial in fighting off SARS-CoV-2 infection [8–10].

The aim of this study was to describe the evolution of vaccination and infection rates in a large and longitudinally followed French cohort of 367 working volunteers, as well as to assess the risk of breakthrough infection based on the careful assessment of both humoral and cellular response post-vaccination.

## Methods

### Participant Recruitment and Data Collection

In this longitudinal cohort study (Covimmune 2), we recruited working volunteers. Participants enrolled were either health care workers (HCWs) or working elsewhere in the public sector (city hall and local administration services) and living in the Alpes-Maritimes area in France. The initial inclusion period lasted from July 2020 to January 2021, with two follow-up visits 6 months (February 2021–July 2021) and 12 months (September 2021–December 2021) later. During the inclusion visit, volunteers enrolled in the study signed a written informed consent in accordance with ethical and legal French policies. During each visit (inclusion, month 6 and month 12), a blood sample was taken and the participants filled in a comprehensive questionnaire about their medical history, as well as a history of COVID-19 infection and vaccination. In order to asses vaccine efficiency, vaccinated participants were asked to fill in a fourth questionnaire (between March 2022 and April 2022), after the Delta-Omicron variant wave of contaminations in early 2022.

The study protocol conformed to the ethical guidelines of the 1975 Declaration of Helsinki and was reviewed and approved by a local institutional review committee (NCT04429594).

### Humoral SARS-CoV-2 Response

Humoral response was assessed at all three time points, by measuring immunoglobulin (Ig) G antibodies against SARS-CoV-2 spike protein (SP) and against nucleocapsid protein (NP) using EUROIMMUN enzyme-linked immunosorbent test (ELISA) kits Anti-SARS-CoV-2 ELISA IgG and Anti-SARS-CoV-2 NCP ELISA (IgG), respectively, following the manufacturer’s instructions. The anti-NP ELISA assay was qualitative, while the levels of anti-SP antibodies were expressed in binding antibody units (BAU)/mL with the positivity threshold of 10 BAU/mL. In the graphical representation, all samples below the *Y* axe were given the value of 1 BAU/mL for visualization purposes.

Sera of participants positive for anti-SP IgG antibodies were tested for their neutralization capacity through a surrogate virus neutralization assay against the Wuhan variant SP, the Alpha variant, and the Delta variant, using MSD V-plex SARS-CoV-2 Panel 17 ACE2 Kit, following the manufacturer’s instructions. Briefly, this assay measures the neutralization capacity of antibodies by creating a competition for spike linkage between sample IgG and ACE2. Results were expressed in units/mL, where 1 unit/mL corresponds to neutralizing activity of 1 µg/mL monoclonal antibody to SARS-CoV-2 SP, as defined by the manufacturer.

### Cellular SARS-CoV-2 Response

At the third time point (month 12), the cellular response against SARS-CoV-2 was assessed using an interferon gamma (IFNγ) Release Immunoassay (IGRA) kit QuantiFERON SARS-CoV-2 (Qiagen). Containing over 30 sequences of different SP domains combined with MHC I and II, the coating in the incubation tube activates CD4 + and CD8 + T cells. One milliliter of whole blood was collected in lithium heparin tubes, then incubated with the mixtures of SARS-CoV-2 antigens as well as negative and positive controls of activation, within 8 h after sampling, at 37 °C for 16 h. The plasma collected was frozen until IFNγ quantification by ELISA (QuantiFERON Human IFNγ, Qiagen), and freeze–thaw cycles were minimized to preserve the quality of samples.

### General T Cell and Innate Immune Cell Response

At the third time point (month 12), the capability of overall T cell response and innate immune response was tested using anti-CD3 (for T cell activation) and TLR 7/8 agonist (as an innate immune cells stimulant) using QuantiFERON MONITOR beads (Qiagen). One milliliter of whole blood was collected in lithium heparin tubes, then incubated with one bead for 16 h at 37 °C after agitation. Plasma was stored at − 80 °C until IFNγ released quantification by ELISA (QuantiFERON Human IFNγ, Qiagen), and freeze–thaw cycles were minimized to preserve the quality of samples.

### Group Criteria and Timeline to Vaccination

According to the vaccination and infection status, four groups can be created: vaccinated and naive to infection, vaccinated and previously infected, unvaccinated and infected, and unvaccinated and naive to infection.

For the vaccinated and naive to infection group, the criteria of selection were (i) the absence of a positive PCR or antigenic SARS-CoV-2 test based on the questionnaire covering the time before and during the study, (ii) the absence of anti-SARS-CoV-2 SP IgG antibodies before vaccination, (iii) the absence of anti-NP antibodies after vaccination based on a serological test performed at all three time points and (iv) had received the two doses of SARS-CoV-2 vaccine. Subjects who had received a third dose of the vaccine or who got infected  after vaccination until the follow-up visit at month 12 were excluded from the group. Subjects classified as vaccinated and previously infected (i) declared a positive PCR or antigenic SARS-CoV-2 test before or during the study, (ii) displayed anti-SP IgG antibodies before vaccination or (iii) anti-NP antibodies after vaccination when symptoms appeared before vaccination and (iv) had received at least one dose of a SARS-CoV-2 vaccine.

Subjects considered unvaccinated and naive to infection were identified with (i) the absence of a positive PCR or antigenic SARS-CoV-2 test based on the questionnaire covering the time before and during the study, (ii) the absence of anti-SARS-CoV-2 SP IgG antibodies at all time points and (iii) no vaccination against SARS-CoV-2. Subjects classified as unvaccinated and infected (i) declared a positive PCR or antigenic SARS-CoV-2 test before or during the study, (ii) displayed anti-SP IgG antibodies in at least one time point and (iii) have no vaccination against SARS-CoV-2.

Vaccination and infection dates were collected with the questionnaire. The participants were vaccinated according to their eligibility for vaccination at the time of vaccine rollout in France, meaning that they were not vaccinated at a certain time point after the initiation of the study. Consequently, the time between the sample collection and vaccination was unique for each participant. In order to manage the delay between the sampling time point and the vaccination, we used the vaccination date and the sampling dates to calculate days between the two events and create a timeline. The time between the vaccination and the next sampling point was calculated and placed on the timeline relative to the vaccination.

### Statistical Analyses

Quantitative variables are presented as mean and standard deviation or median and interquartile range depending on whether the data has a Gaussian distribution or not. Qualitative variables are presented as numbers and percentages. Shapiro–Wilk and D’Agostino and Pearson tests were used to assess the normality of the variables. Continuous data were compared using the Student *t*-test or Mann–Whitney test as appropriate. The nonlinear regression was plotted using least squares fit with a confidence level of 95% and without fitting any subzero *X* values. Qualitative variables were compared using Pearson chi-squared test or Fisher’s exact test in case of small samples. Multivariate logistic regression models were used to investigate independent infection predictive factors and bad vaccine responder’s predictive factors. Variables with a *p*-value ≤ 0.2 in the univariate analysis were included in the multivariate model. Statistical analyses were performed using GraphPad Prism 8 (GraphPad Software, Inc., San Diego, CA) and SAS Enterprise Guide 7.1 for multivariate analysis. All comparisons were two-tailed, and *p*-values < 0.05 were considered significant.

## Results

### Participants’ Characteristics

**Fig. 1:**
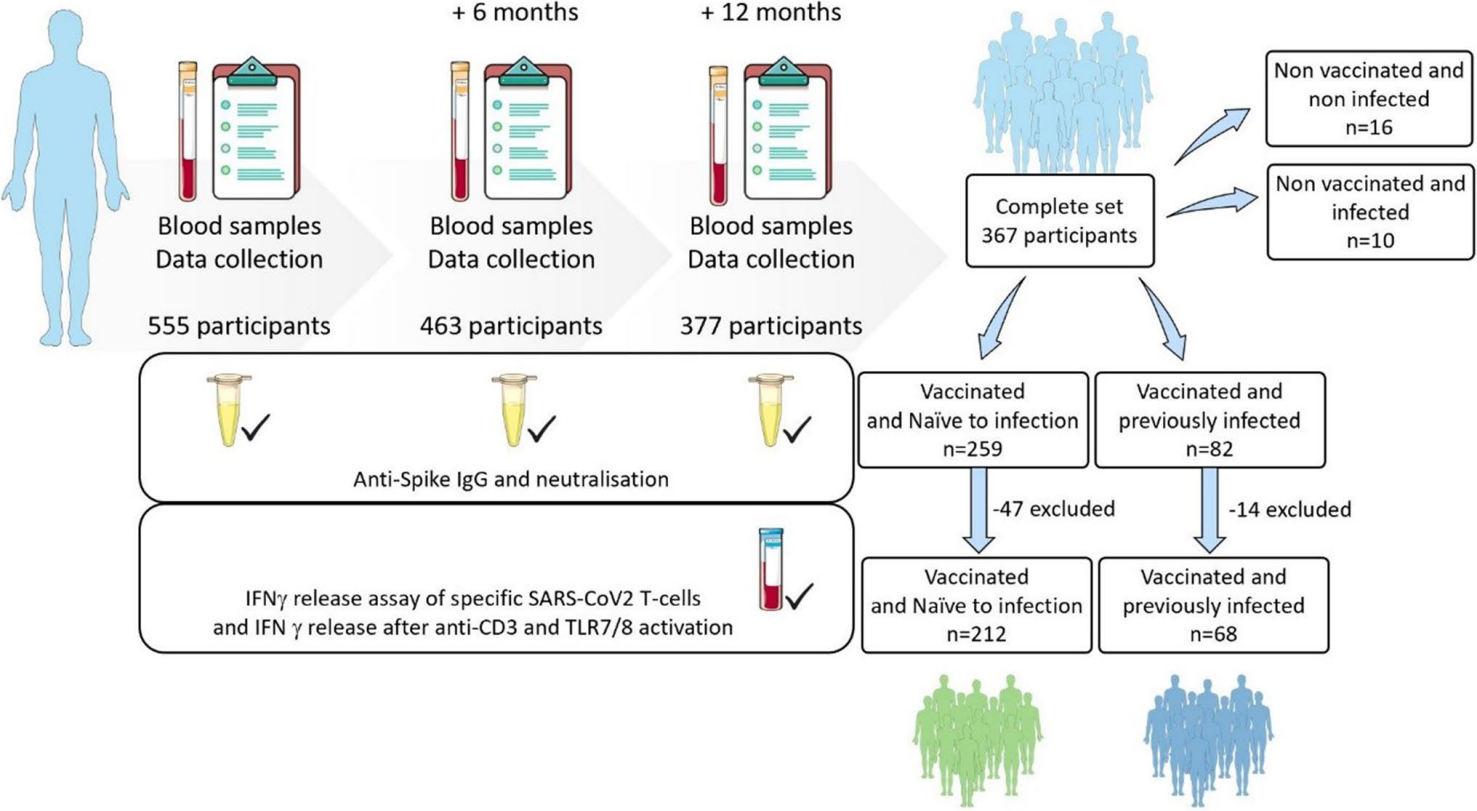
Flowchart of the Covimmune 2 study. Blood samples and data were collected at three time points (month 0, month 6 and month 12) starting in July 2020. At month 0 (*n* = 555) and month 6 (*n* = 463), only B cell response was assessed. For month 12, both B cell and T cell responses were measured. At the end of month 12 (December 2021), 367 participants had a complete set of data for the three time points. According to history of vaccination and SARS-CoV-2 infection, participants were classified as vaccinated and naive to infection (*n* = 212, 47 excluded), vaccinated and previously infected (*n* = 68, 14 excluded), non-vaccinated and non-infected (*n* = 16) and non-vaccinated and infected (*n* = 10)

Of the 555 participants initially included in the study, 463 (83.4%) completed their first follow-up visit at month 6, and 377 (67.9%) completed their second follow-up visit at month 12. Of these, 367 participants had the complete set of three time points and were included in the final dataset (Fig. 1). Among these, 280 vaccinated participants meet the group criteria and 190 (67.9%) of these completed the fourth questionnaire.

Demographic and clinical characteristics of the final dataset of 367 participants who completed all three follow up visits are shown in Table 1. The median age was 51 years at the end of the study, 80 (21.8%) were men and 287 women (78.2%), and 162 (44.1%) participants had at least one comorbidity. Ninety-three of 367 participants (25.3%) were HCWs, and 274 (74.7%) worked in other areas of the public sector.

**Table 1:** Characterization of the 367 participants at each time point

	0 months	6 months	12 months
*n*	367	367	367
Female (*n*, %)	287 (78.2%)	287 (78.2%)	287 (78.2%)
Male (*n*, %)	80 (21.8%)	80 (21.8%)	80 (21.8%)
Age (years), median (IQR)	50 (40; 56)	50 (40; 57)	51 (41; 57)
BMI (ratio), median (IQR)	23.7 (21.5; 27.3)	24.0 (22.0; 27.0)	23.7 (21.8; 27.0)
Comorbidities (*n*, %)	162 (44.1%)	162 (44.1%)	162 (44.1%)
HCWs (*n*, %)	93 (25.3%)	93 (25.3%)	93 (25.3%)
New infections (*n*, %)	34 (9.3%)	40 (10.9%)	18 (4.9%)
Total number of infected (*n*, %)	34 (9.3%)	74 (20.2%)	92 (25.0%)
Time between infection and sampling (days), median (IQR)	68.5 (33.3; 164.5)	258.5 (226.5; 374.3)	387.5 (263.8; 479.8)
Vaccinated (*n*, %)	0	245 (66.8%)	341 (92.9%)
Unvaccinated (*n*, %)	367 (100%)	122 (33.2%)	26 (7.1%)
Number of vaccine doses (*n*, %)	0 (367, 100%)	0 (122, 33.2%)	0 (26, 7.1%)
		1 (100, 27.2%)	1 (60, 16.3%)
		2 (145, 39.5%)	2 (244, 66.5%)
		3 (0)	3 (37, 10.1%)
Vaccinated and naive to infection (*n*, %)	–	208 (56.7%)	259 (70.7%)
Vaccinated and infected (*n*, %)	–	37 (10.0%)	82 (22.3%)
Unvaccinated and infected (*n*, %)	34 (9.3%)	38 (10.4%)	10 (2.7%)
Unvaccinated and naive to infection (*n*, %)	333 (90.7%)	84 (22.9%)	16 (4.4%)
Time between vaccination and sampling (days), median (IQR)	–	39 (21; 75)	188 (140; 230)
Anti-SP IgG (BAU/mL), median (IQR)	59.5 (33.75; 165.5)	278.5 (94; 918.5)	248 (108.8; 734)
Anti-N IgG positivity (*n*, %)	–	30 (40.5% of infected)	26 (28.3% of infected)
Neutralization of Wuhan variant, median (IQR)	2.6 (1.4; 4.3)	7.4 (2.4; 16.6)	6.4 (2.5; 14.0)
Neutralization of Alpha variant, median (IQR)	2.2 (1.2; 3.5)	6.0 (1.9; 14.9)	5.1 (2.1; 12.8)
Neutralization of Delta variant, median (IQR)	2.6 (1.1; 3.9)	5.9 (1.9; 14.5)	5.5 (2.2; 13.5)
SARS-CoV-2 specific T cell response (IU/mL), median (IQR)	–	–	0.41 (0.22; 0.9)

*N* number, *IQR* interquartile range, *HCW* health care workers, *Anti-SP* anti-Spike, *IgG* immunoglobulin G, *Anti-N* anti-nucleocapsid

**Fig. 2:**
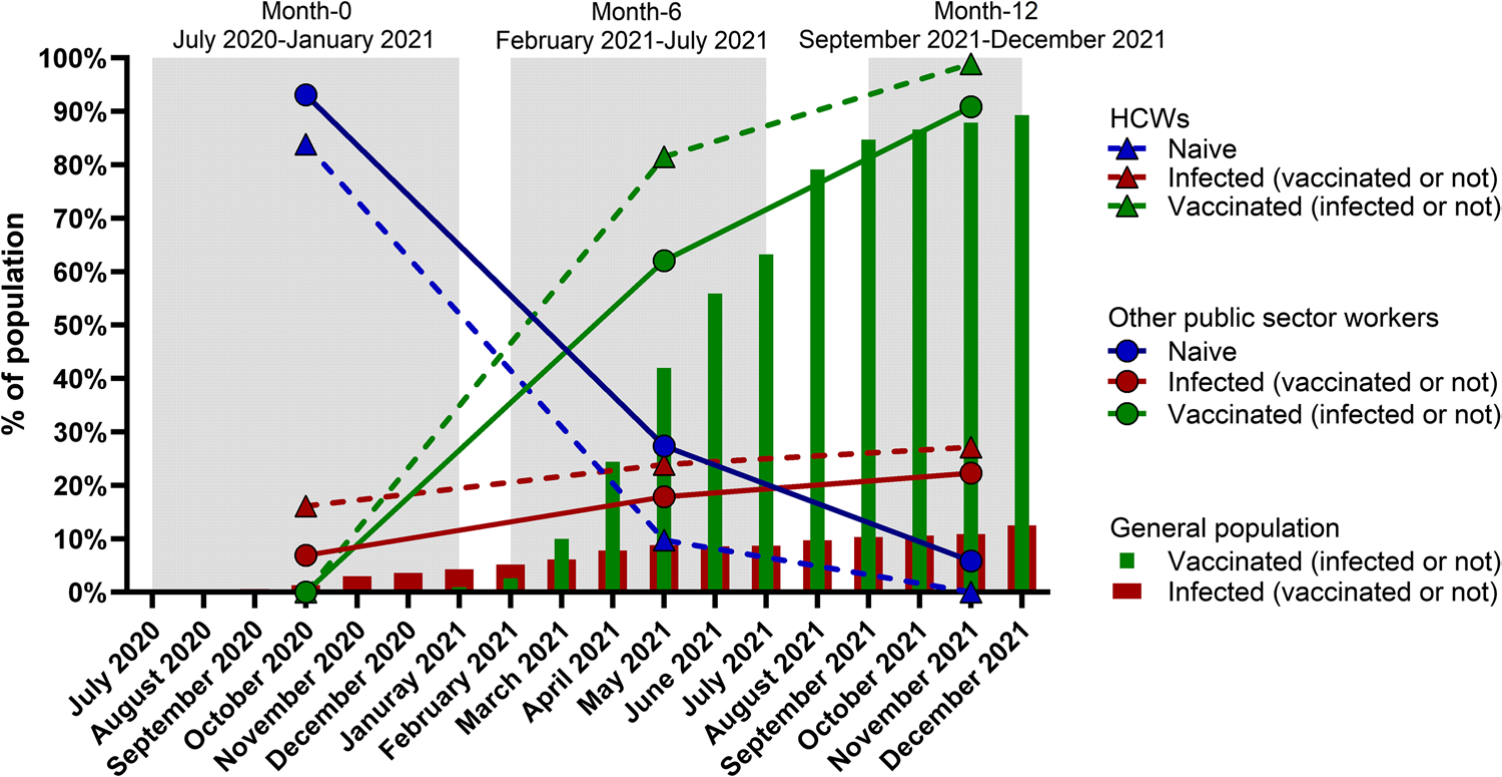
The evolution of vaccination and infection rates in the study cohort (*n* = 367) from July 2020 to December 2021. Relative infection and vaccination rates for each time point (month 0, month 6, month 12, shaded in grey) are shown for each group of participants: HCWs (*n* = 93) and workers in other areas of the public sector (*n* = 274), and compared to the monthly infection and vaccination rates of the general French population. HCWs, health care workers

At baseline visit between July 2020 and January 2021, when no vaccination was available, 34 of 367 (9.3%) participants had a history of infection with SARS-CoV-2 as demonstrated by a positive PCR or antigenic test and/or positive serological test for anti-SP (Table 1 and Fig. 2). At the first follow-up visit at month 6 between February 2021 and July 2021, 74 of 367 (20.2%) participants had a history of SARS-CoV-2 infection, 245 (66.8%) were vaccinated with at least one dose of the SARS-CoV-2 vaccine, and 84 (22.9%) were naive to both infection and vaccination. At the second follow-up visit at month 12 between September 2021 and December 2021, 92 of 367 (25.0%) participants had a history of SARS-CoV-2 infection, 341 (92.9%) were vaccinated with at least one dose of the SARS-CoV-2 vaccine, and 16 (4.4%) were naive to both infection and vaccination. The rate of new infections decreased at the third time point with 34 (9.3%), 40 (10.0%) and 18 (4.9%) new infection at month 0, month 6 and month 12, respectively. HCWs had higher rates of infection in comparison to the participants working in other areas of the public sector (16.1% vs 6.9% at month 0; 23.9% vs 17.9% at month 6 and 27.2% vs 22.3% at month 12 for HCWs vs other public sector, respectively), likely due to the increased contact with COVID-19 patients. Similarly, their rate of vaccination was higher than in the public sector due to COVID-19 vaccine obligation in French hospitals (81.5% vs 62.0% at month 6 and 98.9% vs 90.9% at month 12 for HCWs vs another public sector, respectively). Most participants (87%) received a mRNA vaccine (data not shown). In order to compare the infection and vaccination rates of study participants to the general French population, we extracted infection and vaccination rates from online public data repositories data.gouv.fr and ourworldindata.org. In comparison to the general population, the study participants had a higher rate of infection and of vaccination than the general population at any given time period (month 0, month 6 and month 12; Fig. 2).

Considering the vaccination and infection status, participants were classified in four groups: vaccinated and naive to infection (*n* = 259, 70.6%), vaccinated and previously infected (*n* = 82, 22.3%), unvaccinated and naive to infection (*n* = 16, 4.4%), and unvaccinated and infected (*n* = 10, 2.7%). Due to number of participants in each group, only the two groups of vaccinated individuals were analyzed. Among vaccinated and naive to infection, 47 individuals were excluded since they had received a third injection before month 12 or their samples collected were between dose one and two of vaccination. Only individuals with at least one sample before and after two doses were included in the analysis. In the group of vaccinated and previously infected individuals, 14 were excluded due to uncertainty of infection occurrence before or after vaccination.

### Humoral Response

**Fig. 3:**
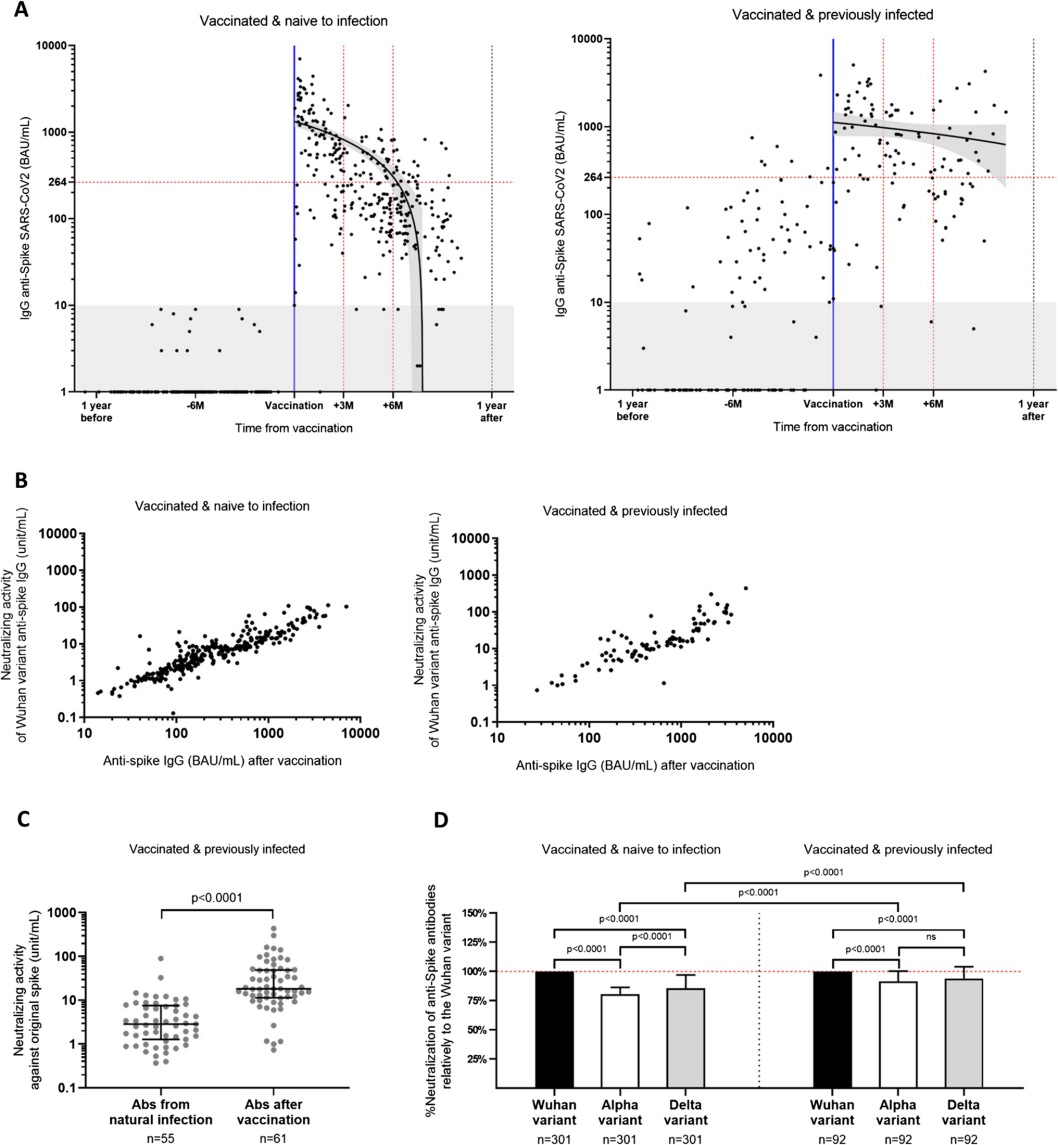
SARS-CoV-2 B cell response evolution in vaccinated individuals naive to infection or previously infected. **A** The three samples collected per participant were placed in a timeline according to their distance in days from the second dose of vaccination. After vaccination, anti-Spike (SP) IgG antibodies rapidly declined after 3 months in participants naive to infection (*n* = 212, *p* < 0.0001, Spearman *r* =  − 0.62). In vaccinated and previously infected participants (*n* = 68), anti-SP IgG levels significantly increased after vaccination and were more stable over time (*p* = 0.018, Spearman *r* =  − 0.22). **B** The neutralization capacity of anti-SP IgG was strongly correlated to the antibody levels in both vaccinated and naive to infection and vaccinated and previously infected (*p* < 0.0001, Spearman *r* = 0.9; *p* < 0.0001, Spearman *r* = 0.86, respectively). **C** In previously infected individuals, the neutralization capacity of anti-SP IgG antibodies significantly increased after vaccination (*p* < 0.0001). **D** Neutralization for the Alpha and Delta variants was compared in percentage to the Wuhan variant. Both groups had a stronger neutralization capacity to the original Wuhan variant than for the Alpha and Delta variants. There was no difference in the neutralization between the Alpha and Delta variants in vaccinated and previously infected individuals. *p < 0.05, **p < 0.005, ***p < 0.0005, ****p < 0.00005

In the group of individuals who were vaccinated and naive to infection, antibody levels before vaccination were below the threshold of positivity. Within 3 months following vaccination, only two of 116 individuals (1.7%) with a sample in this time range had no detectable SARS-CoV-2 anti-SP IgG antibodies, while 31 of 116 individuals (26.7%) remained below the protective threshold of 264 BAU/mL determined previously for the Alpha variant [11]. There were significant differences in age and comorbidities between individuals above and below the protective threshold. Individuals with SARS-CoV-2 anti-SP IgG antibodies above 264 BAU/mL were younger (47 (IQR, 39; 56) vs 50 (42; 57) years, *p* = 0.045) and presented less comorbidities (50 (36.5%) individuals with at least one comorbidity vs 82 (49.7%), *p* = 0.027). The sex ratio and HCWs’s proportion were the same between the groups (Table 2). Between month 3 and month 6 post-vaccination, anti-SP IgG antibody levels started to significantly decrease. Sixty-two of 96 individuals (64.6%) were below the protective threshold of 264 BAU/mL. Over 6 months after vaccination, antibody levels rapidly decreased as 87 of 107 individuals (81.3%) were below the protective threshold of 264 BAU/mL. We found a significant inverse correlation between time post-vaccination and anti-SP IgG antibody levels (*p* < 0.0001, Spearman *r* =  − 0.62) (Fig. 3A left), i.e., post-vaccination time was longer for participants with sub-threshold anti-SP antibody levels compared to those with protective levels (176 and 73 days, respectively) (*p* < 0.0001) (Table 2).

**Table 2:** Characterization of individuals above and below 264 BAU/mL in the two groups: vaccinated and naive to infection and vaccinated and previously infection

Characteristics	Vaccinated and naive to infection (VNI)				Vaccinated and previously infected (VPI)				*p*-value^1^		
	IgG Spike < 264 BAU/mL	IgG Spike > 264 BAU/mL	*p*-value^1^	Total	IgG Spike < 264 BAU/mL	IgG Spike > 264 BAU/mL	*p*-value^1^	Total	Total: VNI vs VPI	< 264 BAU/mL: VNI vs VPI	> 264 BAU/mL: VNI vs VPI
*n*	165	137		212	27	68		68			
M/F	31/134	22/115	0.76	40/172	4/23	18/30	0.038*	17/51	0.3	0.65	0.0038**
Age (years), median (IQR)	50 (42; 57)	47 (39; 56)	0.045*	49 (41; 56)	51 (37; 56)	51 (39; 57)	0.59	51 (39.3; 57)	0.85	0.55	0.33
BMI (ratio), median (IQR)	23.6 (21.8; 26.9)	23.7 (22; 26.4)	0.59	23.6 (21.7; 26.9)	23.4 (21.9; 25.2)	25 (22.3; 28.6)	0.09	24.2 (22.2; 26.6)	0.5	0.54	0.19
Comorbidities (*n*, %)	82 (49.7%)	50 (36.5%)	0.027*	90 (42.5%)	15 (55.6%)	30 (44.1%)	0.37	32 (47.1%)	0.57	0.68	0.36
HCWs (*n*, %)	41 (24.8%)	38 (27.8%)	0.6	51 (24.1%)	11 (40.8%)	21 (30.9%)	0.48	21 (30.9%)	0.27	0.1	0.74
Time between infection and sampling (days), median (IQR)	–	–		–	387 (109; 453)	285 (235.5; 398.3)	0.42	236 (80; 243)	–	–	–
Time between vaccination and sampling (days), median (IQR)	176 (134; 212.5)	73 (32.5; 142.5)	< 0.0001	145 (66; 194)	188 (127; 230)	102 (47.3; 153.8)	0.0021**	112 (47; 196)	0.28	0.65	0.049*
Number of vaccine doses, (*n*, %)	2 (165, 100%)	2 (137, 100%)	–	2 (212, 100%)	1 (14, 51.9%); 2 (13, 48.1%)	1 (47, 69.1%); 2 (21, 30.9%)	0.15	1 (43, 63.2%); 2 (25, 36.8%)	–	–	
Anti-SP IgG (BAU/mL), median (IQR)	109 (63; 158.5)	722 (465.5; 1331)	< 0.0001	208 (97; 630.5)	138 (51; 185)	914.5 (460.8; 1617)	< 0.0001	473 (185; 1396)	< 0.0001	0.3	0.19
Neutralization of Wuhan variant, median (IQR)	2.5 (1.5; 4.5)	12.8 (8.2; 23.6)	< 0.0001	6.3 (2.4; 12.8)	4.8 (2.1; 8.8)	18.2 (11.7; 51.2)	< 0.0001	15 (6.6; 34.4)	< 0.0001	0.009**	0.0013**
Neutralization of Alpha variant, median (IQR)	2.1 (1.2; 3.5)	11.1 (6.3; 20.6)	< 0.0001	4.8 (2; 10.5)	4.2 (1.8; 9.4)	17.2 (11.2; 47.8)	< 0.0001	13.5 (6.1; 34.3)	< 0.0001	0.0027**	< 0.0001
Neutralization of Delta variant, median (IQR)	2.3 (1.5; 4)	11.6 (6.7; 21.2)	< 0.0001	5.1 (2.8; 10.8)	4.4 (1.9; 8.5)	19.6 (11.7; 51.8)	< 0.0001	13.8 (6.1; 41.4)	< 0.0001	0.01*	< 0.0001
SARS-CoV-2 specific T cell response (IU/mL), median (IQR)	0.21 (0.09; 0.42)	0.29 (0.16; 0.9)	0.0023**	0.23 (0.1; 0.54)	0.2 (0.095; 0.68)	0.65 (0.31; 1.9)	0.0014**	0.52 (0.23; 1.23)	< 0.0001	0.72	0.0018**

*N* number, *IQR* interquartile range, *HCW* health care workers, *Anti-SP* anti-Spike, *IgG* immunoglobulin G, *Anti-N* anti-nucleocapsid

*p < 0.05, **p < 0.005, ***p < 0.0005, ****p < 0.00005

^1^Mann-Whitney test for quantitative variables; Fisher’s exact test for qualitative variables. All tests are two-sided with statistical significance *p* < 0.05

In the group of previously infected individuals, only eight of 52 participants (15.4%) had anti-SP IgG antibody levels above the protective threshold of 264 BAU/mL prior to vaccination (Fig. 3A right), independently of the time elapsed since infection (data not shown). Anti-SP IgG antibody levels significantly increased post-vaccination (*p* < 0.0001) and were more stable over time as only 20 of 59 (33.9%) individuals were below 264 BAU/mL of anti-SP IgG antibodies 3 months after vaccination in comparison to 149 of 203 (73.4%) vaccinated individuals naive to infection (*p* < 0.0001, Table 2). The inverse correlation between number of days since vaccination and anti-SP IgG antibody levels was significantly lower in vaccinated and previously infected individuals than in vaccinated and previously uninfected individuals (*p* = 0.018, Spearman *r* =  − 0.22) (Fig. 3A). Despite this, within 3 months after vaccination, there was no significant difference in anti-SP IgG antibody levels between naive or previously infected individuals. No association was found in vaccinated and previously infected individuals between anti-SP IgG levels and the number of vaccine doses (data not shown).

In the groups of both vaccinated and naive to infection and vaccinated and previously infected individuals, the neutralization capacity of anti-SP IgG antibodies was correlated to their levels (*p* < 0.0001, Spearman *r* = 0.9; *p* < 0.0001, Spearman *r* = 0.86, respectively) (Fig. 3B). In previously infected individuals, the neutralization capacity of anti-SP IgG antibodies significantly increased after vaccination (*p* < 0.0001) (Fig. 3C). Independently of the variant (Wuhan, Alpha or Delta), vaccinated and previously infected individuals exhibited a higher median neutralization capacity (15.0, 13.5 and 13.8 unit/mL, respectively) than vaccinated individuals naive to infection (6.3, 4.8 and 5.1 unit/mL, respectively) (*p* < 0.0001) (Table 2).

As the neutralization capacity of the Wuhan variant was higher than the neutralization of Alpha and Delta variants, a percentage of neutralization of the Alpha and Delta variants was calculated for each individual in comparison to the Wuhan variant. The neutralization of Alpha variant was significantly lower in comparison to the Wuhan variant in the group of individuals naive to infection (80.7%) and in the group of vaccinated and previously infected individuals (91.5%, *p* < 0.0001). Similar results were obtained for the Delta variant (85.6% relative neutralization in the group of vaccinated individuals naive to infection vs 94.1% in the group of vaccinated and previously infected individuals, *p* < 0.0001) (Fig. 3D). The median neutralization capacity against the Alpha variant in vaccinated and previously infected individuals was 91.5% vs 80.7% in vaccinated individuals naive to infection (*p* < 0.0001). Similarly, vaccinated and previously infected individuals had a 94.1% neutralizing capacity of the Delta variant vs 85.6% in vaccinated individuals naive to infection (*p* < 0.0001) (Fig. 3D).

### Cellular Response

Using IGRA, we measured the SARS-CoV-2-specific T cell response in participants after infection and/or vaccination at the third and last sampling point at month 12 after inclusion in the study. As the cellular response was measured only at month 12, a selection of samples within 9 months after vaccination was performed to normalize the time between vaccination and sampling.

Two hundred and seventy of 367 participants (71.8%) had a positive SARS-CoV-2-specific cellular response. Among vaccinated participants naive to infection, 156 of 203 (76.8%) were positive to the test, in comparison to 61 of 68 (89.7%) vaccinated and previously infected participants (*p* = 0.023). There were no significant differences in age, comorbidities or gender or HCWs between groups (Table 3). There was no difference in the levels of cellular response in vaccinated and previously infected individuals depending on the number of vaccine doses (data not shown). Humoral and cellular responses were positively correlated in both vaccinated and naive to infection (*p* = 0.0011, Spearman’s *r* = 0.37) and in vaccinated and previously infected (*p* = 0.0018, Spearman’s *r* = 0.22) individuals.

**Table 3:** Characterization of individuals displaying a detectable or undetectable SARS-CoV-2 T cell response in the two groups: vaccinated and naive to infection and vaccinated and previously infection

Characteristics	Vaccinated and naive to infection (VNI)			Vaccinated and previously infected (VPI)			
	Undetectable SARS-CoV-2 T cell response	Detectable SARS-CoV-2 T cell response	*p*-value^1^	Undetectable SARS-CoV-2 T cell response	Detectable SARS-CoV-2 T cell response	*p*-value^1^	*p*-value: detectable T cell response VNI vs VPI
*n*	47	156		7	61		
M/F	12/35	26/130	0.2	0/7	17/42		0.06
Age (years), median (IQR)	43 (37; 57)	50 (42; 56)	0.05	48 (37; 57)	51 (40; 57)	0.6	0.9
BMI (ratio), median (IQR)	23.6 (21.4; 26.9)	23.7 (21.8; 26.8)	0.6	23.4 (22.1; 24.2)	25 (22.3; 27.9)	0.1	0.2
Comorbidities (*n*, %)	21 (44.7%)	67 (42.9%)	0.9	3 (42.7%)	27 (44.3%)	> 0.9	0.9
HCWs (*n*, %)	12 (25.5%)	32 (20.5%)	0.5	3 (42.7%)	18 (29.5%)	0.7	0.2
Time between infection and sampling (days), median (IQR)	–	–		387 (287.5; 404.5)	342 (263.3; 443)	> 0.9	–
Time between vaccination and sampling (days), median (IQR)	176 (133; 206)	177 (145.3; 209.8)	0.9	196 (153; 217)	175 (102; 218)	0.4	0.09
Number of vaccine doses, (*n*, %)	2 (51, 100%)	2 (156, 100%)		1 (3, 42.9%); 2 (4, 57.1%)	1 (39, 63.9%); 2 (20, 32.8%)	0.2	–
Anti-SP IgG (BAU/mL), median (IQR)	104 (67; 193)	164 (81.5; 349.3)	0.014*	182 (95; 803)	473 (242.5; 1414)	0.06	< 0.0001
Neutralization of Wuhan variant, median (IQR)	2.8 (1.9; 7.4)	4.5 (2.2; 8.3)	0.057	9 (4; 18)	13.1 (7; 34.8)	0.2	< 0.0001
Neutralization of Alpha variant, median (IQR)	2.2 (1.6; 5.4)	3.5 (1.7; 6.3)	0.09	9.7 (3.7; 15.5)	13.2 (6.6; 35.6)	0.15	< 0.0001
Neutralization of Delta variant, median (IQR)	2.7 (1.7; 5.6)	3.9 (1.8; 6.7)	0.09	8.8 (4; 11.8)	14 (6.7; 42.3)	0.1	< 0.0001
SARS-CoV-2 specific T cell response (IU/mL), median (IQR)	–	0.3 (0.19; 0.71)		–	0.59 (0.27; 1.5)	–	0.0002***

*N* number, *IQR* interquartile range, *HCW* health care workers, *Anti-SP* anti-Spike, *IgG* immunoglobulin G, *Anti-N* anti-nucleocapsid

*p < 0.05, **p < 0.005, ***p < 0.0005, ****p < 0.00005

^1^Mann-Whitney test for quantitative variables; Fisher’s exact test for qualitative variables. All tests are two-sided with statistical significance *p* < 0.05

**Fig. 4:**
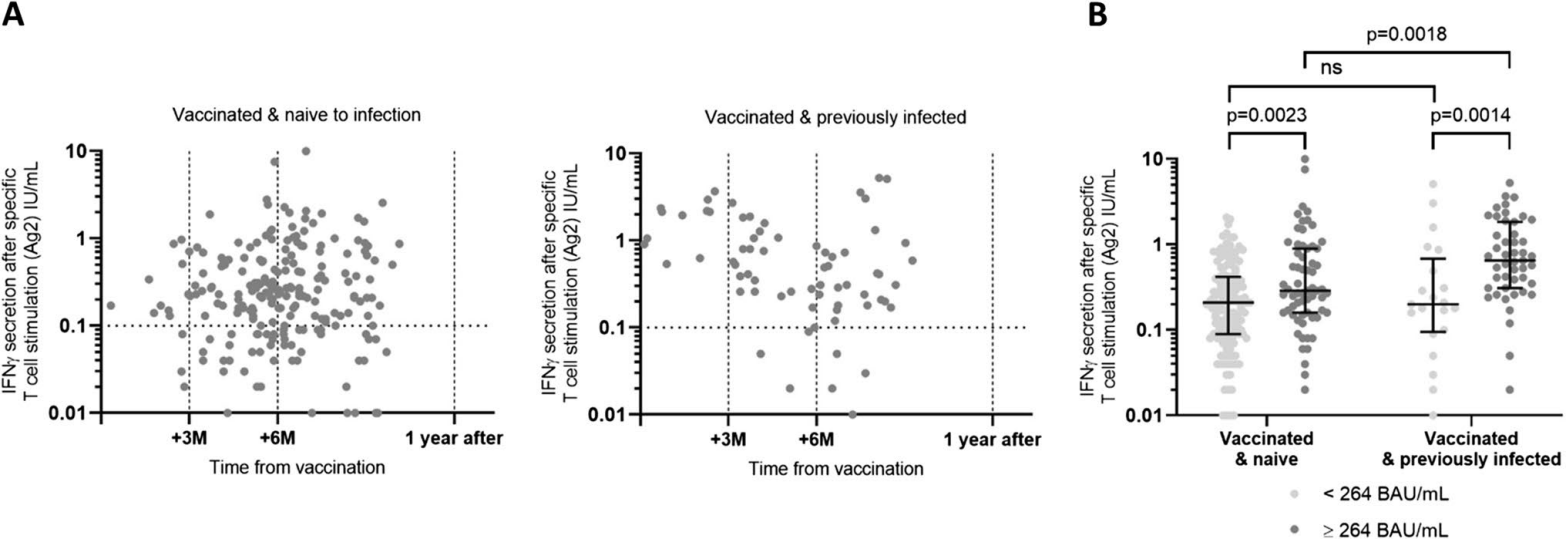
SARS-CoV-2 T cell response evolution in vaccinated individuals naive to infection or previously infected. **A** At month 12 of the study, plasma IFNγ was measured after SARS-CoV-2 specific T cell activation. Samples were placed in a timeline according to their distance in days from the second dose of vaccination. In vaccinated and naive to infection individuals, T cell response was stable over time (*p* = 0.3, Spearman *r* = 0.07). In the vaccinated and previously infected group, a slight decline of the cellular response was observed (*p* = 0.004, Spearman *r* =  − 0.3). **B** Taking in consideration anti-SP IgG levels above or below the threshold of 264 BAU/mL, plasma IFNγ levels were compared between groups. In both groups, plasma IFNγ levels were higher in individuals displaying anti-SP IgG levels above the threshold. In individuals with anti-SP IgG levels above the threshold, median IFNγ levels were significantly higher in vaccinated and previously infected individuals (0.65 IU/mL vs 0.29 IU/mL). *p < 0.05, **p < 0.005, ***p < 0.0005, ****p < 0.00005

Contrary to humoral response, in the group of vaccinated individuals naive to infection, there was no correlation between the level of the cellular response and the time since vaccination (*p* = 0.3, Spearman *r* = 0.07) (Table 3, Fig. 4A left). In the vaccinated and previously infected group, a slight decline of the cellular response was observed corresponding to a significant negative correlation with time since vaccination (*p* = 0.004, Spearman *r* =  − 0.3) (Fig. 4A right). Despite this decline, median values of IFNγ in vaccinated and previously infected individuals with a detectable SARS-CoV-2 T cell response were significantly higher than in vaccinated individuals naive to infection (0.6 IU/mL and 0.3 IU/mL, respectively, *p* = 0.0002) (Table 3).

In the group of vaccinated participants naive to infection, individuals with anti-SP IgG antibody levels below 264 BAU/mL had a median of 0.21 IU/mL of IFNγ vs 0.29 IU/mL in individuals with more than 264 BAU/mL (*p* = 0.0023). In vaccinated and previously infected individuals, IFNγ secretion was 0.20 and 0.65 IU/mL for participants with antibody levels below 264 BAU/mL and above 264 BAU/mL, respectively (*p* = 0.0014). Between the two groups, median IFNγ levels did not differ in individuals with anti-SP IgG levels below 264 BAU/mL (0.21 IU/mL vs 0.20 IU/mL, *p* = 0.7). Above this threshold, vaccinated and previously infected individuals displayed significantly higher median IFNγ levels than vaccinated individuals naive to infection (0.65 IU/mL vs 0.29 IU/mL, respectively, *p* = 0.0018) (Fig. 4B).

### Infection Predictive Factors

In the second part of the study, information about infections occurring between December 2021 and April 2022 was collected and correlated to the prior humoral and cellular response as measured during the third sampling time point at month 12 between September 2021 and December 2021 to measure the performance of these different vaccination schedules. The last sample was used to evaluate the existing immune response level against SARS-CoV-2 just before the new wave of Delta- and Omicron-related contaminations in the beginning of 2022. Of 212 individuals who were vaccinated and naive to infection at month 12, 169 (88.9%) answered the questionnaire, and so this was the only group analyzed. Thirty-five of 169 (20.7%) were tested positive to SARS-CoV-2 between December 2021 and April 2022 presenting either no symptoms (*n* = 5, 14.3%) or mild symptoms without requiring hospitalization (*n* = 30, 85.8%).

**Fig. 5:**
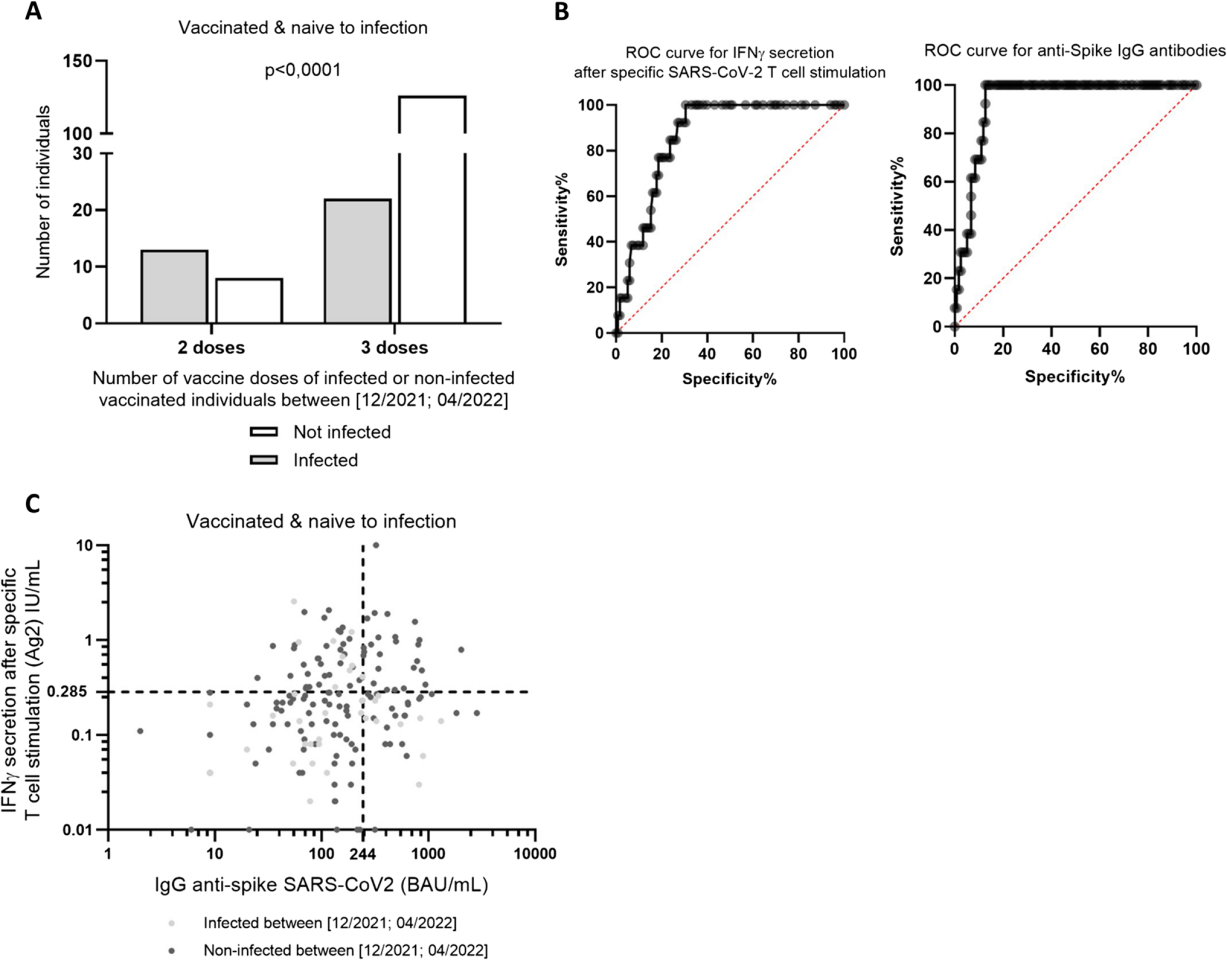
Combining B cell and T cell responses to identify vaccine bad responders in risk of infection. **A** Number of SARS-CoV-2 vaccine doses in individuals who got infected during the infection wave between December 2021 and April 2022 in France. Individuals who had received a booster vaccine (dose 3) got less infected than individuals with only two doses. **B** ROC curves for IFNγ secretion after specific SARS-CoV-2 T cell activation and for anti-SP IgG antibody levels. Defining the threshold of 244 BAU/mL anti-SP IgG antibodies (100.0% sensibility, 78.6% specificity) and 0.285 IU/mL of IFNγ secretion (100% sensibility, 72.1% specificity) where no infected individuals were found. **C** Vaccinated individual’s SARS-CoV-2 immunity (anti-SP IgG and IFNγ secretion) at month 12, just before the wave of contaminations between December 2021 and April 2022. The thresholds of anti-SP IgG ≥ 244 BAU/mL and IFNγ ≥ 0.285 IU/mL create four quadrants (Q). Q4 was define as the good responder’s area with no infection occurrence. Q1–3 area included bad vaccine responders and individuals with a declining immunity. *p < 0.05, **p < 0.005, ***p < 0.0005, ****p < 0.00005

During this period, there were no significant differences between infected and not infected participants in gender ratio, comorbidities nor being HCW (more exposed to the virus). Infected individuals were significantly younger than non-infected individuals (median 44 vs 52 years, respectively, *p* = 0.0026). Time since the last vaccine dose and the questionnaire between the vaccinated individuals who got infected (109 days) and those who did not get infected (96 days) was significantly different (*p* < 0.0001). However, infection happens in median 74 days after the last dose. There was also a difference in the number of vaccine doses, as only 22 of 35 infected individuals (62.9%) had received a third dose vs 126 of 134 individuals (94.0%) who did not get infected over the same period of time (*p* < 0.0001) (Fig. 5A). Vaccinated individuals who got infected tend to have slightly lower IFNγ release after SARS-CoV-2 T cell stimulation after two doses (0.2 IU/mL) than vaccinated individuals who did not get infected (0.3 IU/mL) (*p* = 0.06) (Table 4).

**Table 4:** Characterization of vaccinated and naive to infection individuals who respond to the post-12-month questionnaire

Characteristics	All post-12-months	Not infected post-12-months	Infected post-12-months	*p*-value^1^	
				Univariable	Multivariable
*n*	169	134	35		
M/F	32/137	27/107	5/30	0.6	
Age (years), median (IQR)	50 (42; 57)	52 (43; 58)	44 (35; 52)	0.0026	0.0084
BMI (ratio), median (IQR)	23.5 (21.7; 27)	23.6 (21.7; 27.1)	23.3 (21.2; 26.2)	0.63	
Comorbidities (*n*, %)	67 (39.6%)	55 (41%)	12 (34.3%)	0.6	
HCWs (*n*, %)	38 (22.5%)	26 (19.4%)	12 (34.3%)	0.07	0.25
Time between last dose and questionnaire (days), median (IQR)	–	96 (75.5; 107.3)	74 (39; 130)	0.0001	0.14
Time between vaccination and sampling (days), median (IQR)	181 (144; 224)	181.5 (144; 223.3)	180 (139; 238)	0.95	
Number of vaccine doses before sampling (*n*, %)	2 (169, 100%)	2 (134, 100%)	2 (35, 100%)	–	
Current number of vaccine doses (*n*, %)	–	3 (126, 94%)	3 (22, 62.9%)	< 0.0001	0.28
Anti-SP IgG (BAU/mL), median (IQR)	147 (70; 320.5)	149.5 (70.8; 331)	129 (62; 259)	0.5	
Neutralization of Wuhan variant, median (IQR)	3.9 (1.9; 7.9)	4.1 (2; 7.9)	3.8 (1.7; 7)	0.7	
Neutralization of Alpha variant, median (IQR)	3.3 (1.5; 6)	3.4 (1.5; 6.2)	3.2 (1.5; 5.4)	0.7	
Neutralization of Delta variant, median (IQR)	3.1 (1.6; 6.4)	3 (1.5; 6.5)	3.5 (1.8; 5.4)	0.9	
SARS-CoV-2 specific T cell response (IU/mL), median (IQR)	0.23 (0.1; 0.53)	0.25 (0.12; 0.58)	0.15 (0.08; 0.32)	0.06	0.04

*N* number; *IQR* interquartile range; *HCW* health care workers; *Anti-SP* anti-Spike; *IgG* immunoglobulin G; *Anti-N* anti-nucleocapsid

*p < 0.05, **p < 0.005, ***p < 0.0005, ****p < 0.00005

^1^Mann-Whitney test for quantitative variables; Fisher’s exact test for qualitative variables. All tests are two-sided with statistical significance *p* < 0.05. To identify infection and bad vaccine responder’s predictive factors, all variables with *p* < 0.15 in unadjusted analysis were used for multivariable logistic regression models

Multivariable analysis of these factors showed than when an individual increases his age of 1 year, his risk of infection decreases by 1.5% (*p* = 0.0084). In the same way for each additional unit of IFNγ secreted after specific T cell activation, the risk of infection decreases by 73.5% (*p* = 0.04, Table 4).

### Characterization of Bad Vaccine Responders

Crossing data from infections during the Delta-Omicron wage, with both T cell and B cell responses after two doses of SARS-Cov-2 vaccines, we performed a ROC curve analysis for the levels of anti-SP IgG antibodies and SARS-CoV-2-induced IFNγ secretion. A threshold of 244 BAU/mL anti-SP IgG antibodies (100.0% sensibility, 78.6% specificity) and 0.285 IU/mL of IFNγ secretion (100% sensitivity, 72.1% specificity) was established (Fig. 5B) defining four quadrants (Q). This allows to distinguish an area of values where no infections were found (Q4) from an area where individuals got infected (Q1–3) (Fig. 5C). Individuals in Q4 area were considered good vaccine responders, and individuals in Q1–3 were bad vaccine responders or individuals with a declining SARS-CoV-2 immunity. No differences in the number of infections between Q1 (9 of 40, 22.5%), Q2 (17 of 74, 23.0%), and Q3 (9 of 30, 30.0%) were observed.

There were no statistical differences in gender, age or the number of vaccine doses between the two areas. Time between sampling and the second vaccine dose was higher in individuals in the Q1–3 area than in individuals in the Q4 area (186.5 vs 160.0 days, respectively, *p* = 0.0171). In the Q1–3 area, 62 of 144 (43.1%) individuals displayed at least one comorbidity vs only five of 25 (20.0%) in the Q4 area (*p* = 0.03). As there was a difference in comorbidities among the two areas, we measured the general capability of T cells and innate immunity cells to secrete IFNγ after anti-CD3 and TLR 7/8 agonist stimulation. There were no statistical differences of IFNγ secretion capability between individuals from Q4 and Q1–3 (100.0 vs 165.0 IU/mL, respectively, *p* = 0.39) (Table 5).

**Table 5:** Characterization of individuals in Q4 versus Q1–3

Characteristics	Q4	Q1-3	*p*-value^1^: Q4 vs Q1–3	
			Univariable	Multivariable
*n*	25	144		
M/F	6/19	28/116	0.8	
Age (years), median (IQR)	48 (40.5; 58.5)	50.5 (42.0; 57.0)	0.5	
BMI (ratio), median (IQR)	23.1 (21.5; 27.5)	23.6 (21.7; 26.8)	1.0	
Comorbidities (*n*, %)	5 (20.0%)	62 (43.1%)	0.03	0.03
HCWs (*n*, %)	2 (8.0%)	36 (25.0%)	0.07	0.18
Time between last dose and questionnaire (days), median (IQR)	90.0 (76; 108)	101.0 (86.0; 112.5)	0.22	
Time between D2 and sampling (days), median (IQR)	160.0 (107.5; 203.5)	186.5 (154.0; 225.0)	0.02	0.1
Current number of vaccine doses, median (IQR)	3 (22, 88.0%)	3 (125, 86.8%)	1.0	
Anti-SP IgG (BAU/mL), median (IQR)	414.0 (311.0; 766.0)	124.0 (67.0; 221.0)	< 0.0001	
Neutralization of Wuhan variant, median (IQR)	8.5 (5.5; 12.7)	2.8 (1.5; 6.3)	< 0.0001	
Neutralization of Alpha variant, median (IQR)	6.6 (5.3; 10.2)	2.3 (1.2; 4.8)	< 0.0001	
Neutralization of Delta variant, median (IQR)	6.9 (5.8; 11.9)	2.6 (1.3; 5.1)	< 0.0001	
SARS-CoV-2 specific T cell response (IU/mL), median (IQR)	0.79 (0.49; 1.1)	0.19 (0.08; 0.33)	< 0.0001	
Unspecific T cell stimulation (IU/ml), median (IQR)	100.0 (20.0; 245.0)	165.0 (26.0; 378.0)	0.39	

Participants were divided into four quartiles depending on their level of anti-SP IgG (above or below the threshold of 244 BAU/mL) and IFNγ (above or below the threshold of 0.285 IU/mL). None of the participants with both values above the threshold (Q4) was infected after M12, while in the Q1, Q2, and Q3, the participants had values of either anti-SP IgG and/or IFNγ below the threshold, and some of them got infected after M12

*N* number; *IQR* interquartile range; *HCW* health care workers; *Anti-SP* anti-Spike; *IgG* immunoglobulin G; *Anti-N* anti-nucleocapsid

*p < 0.05, **p < 0.005, ***p < 0.0005, ****p < 0.00005

^1^Mann-Whitney test for quantitative variables; Fisher’s exact test for qualitative variables. All tests are two-sided with statistical significance *p* < 0.05. To identify infection and bad vaccine responder’s predictive factors, all variables with *p* < 0.15 in unadjusted analysis were used for multivariable logistic regression models

Multivariable analysis of the factors showed that only the presence of comorbidities explained the difference among individuals in the two areas (*p* = 0.03). An individual presenting at least one comorbidity was 3.2 times more likely to be in the Q1–3 area (Table 5). In our cohort, the most common comorbidities were active smoking and alcohol drinking (30 of 62, 48.5%, for both). Ten of 62 individuals (16.1%) with comorbidities had an autoimmune disease, 14 (22.6%) had high blood pressure, four (6.5%) had cancer, three (4,8%) had chronic bronchitis and two (3.2%) had diabetes.

## Discussion

In this study, we described the evolution of humoral and cellular response to SARS-CoV-2 infection and vaccination in a longitudinally followed cohort of 367 working volunteers from Alpes-Maritimes area, France, spanning from the beginning of COVID-19 pandemic in May 2020 to the spread of the Omicron variant in spring 2022.

In comparison to national averages, our cohort was representative of general French population in terms of infection and vaccination rates at any given time point of the follow-up, with some notable differences. First, due to the inclusion of HCWs in our cohort who had facilitated access to SARS-CoV-2 vaccine at the beginning of national vaccination rollout and for whom the vaccination became mandatory in 2021 [12], our cohort showed slightly higher vaccination rates than the general population. Second, we were able to identify more infected patients in our cohort than the average national infection rate, likely due to the anti-N SARS-CoV-2 antibodies allowing to detect asymptomatic infections in vaccinated individuals.

Exposure to SARS-CoV-2 by either natural infection or vaccination elicits a strong immunological innate and adaptive immune response [2]. The innate immunity cells provide a first line defence against the virus and are important to activate adaptive immune response. On the adaptive immunity side, both humoral B cell-mediated immunity and cellular T cell-mediated immunity are crucial in viral clearance after infection and protection from breakthrough infection after vaccination [2, 8] and have been extensively studied in order to improve vaccine efficiency and to identify biomarkers of protection against infection.

Numerous studies have demonstrated the protective effect of anti-SARS-CoV-2 antibodies produced after infection or vaccination against future infections. However, the level of neutralizing antibodies decreases over time [13–15], as was also the case in our cohort. Here, we showed that the waning of humoral response was faster in vaccinated but naive to infection individuals than in vaccinated and previously infected individuals. In addition, individuals who have been in contact with the virus exhibited higher median levels of anti-SP IgG antibodies but most importantly a higher neutralization capacity independently from median anti-SP IgG levels. Indeed, the hybrid immunity after two different stimuli (a virus and a vaccine) is associated with a higher level of anti-SARS-CoV-2 antibodies that persist longer post-vaccination and that are more efficient than in the individuals who have only been vaccinated without prior infection [14]. In line with these results, heterologous vaccine regimen seems to induce a more robust immune response and offer a better protection against future infections than homologous vaccination with the same vaccine [16–18]. In conclusion, diversification of viral stimuli, either by heterologous vaccination or by vaccination combined with previous infection, seems to confer a more robust immune response and a better protection against future infection than the repeated exposure to the same stimulus.

Contrary to humoral response, T cell response seems to be more persistent and may remain at the same level several months post-vaccination or post-infection [9], as we have also observed in our cohort where no significant decline in IFNγ production was observed over time in vaccinated individuals naive to infection. In vaccinated and previously infected individuals, we found a slight decline in IFNγ levels over time, although very few individuals had an undetectable T cell response and median levels of IFNγ secretion were still higher in previously infected individuals than in the vaccinated and naive to infection group. These result together with the B cell response, supporting the idea of a stronger immunity in individuals whom have been in contact with the virus in addition to vaccination.

In light of new emerging variants that seem to escape antibody neutralization [6, 19], T cell response should be measured systematically as, contrary to humoral response, seems to be independent of the variant [9]. The efficiency of the T cell response is of particular importance for patients with autoimmune diseases receiving immunosuppressive treatment to block their humoral response, or in organ transplant recipients, who have a weaker functional B cell response after vaccination, but their T cell response may nevertheless provide sufficient protection against infection [20–22].

In addition, there was no significant difference in either humoral or cellular response between the two groups in the first 3 months after vaccination. These two factors seem to support the recommendation of a booster dose in individuals naive to infection 3 months after the last vaccination. Establishing a threshold of immunity has a biological and economical importance. Targeting individuals in need of a booster vaccine dose would reduce the number of doses necessary for each season while avoiding immunity overactivation side effects in individuals with a present high immunity. Our data suggests that both humoral and cellular responses are necessary to categorize a vaccine immunity high enough to prevent infection. We found no infection occurring in individuals with both anti-SP IgG above 244 BAU/mL and a SARS-CoV-2 specific-T cell’s IFNγ secretion over 0.285 IU/mL after two doses of vaccine. We categorized these individuals as good vaccine responders not needing a vaccine booster while remaining above the thresholds. However, as immunity fades with time and individuals above the thresholds were closer to their vaccination, we hypothesize that in the future they are going to migrate to the Q1–3 area of the graph, although the presence of comorbidities was the factor that revealed to truly determined whether an individual is a good responder or not. Comorbid individuals are known to develop less efficient immunity to vaccines, and we have already shown the benefit of a booster dose in this group  [20]. Also interesting, our threshold of humoral immunity (244 BAU/mL) is relatively closed to the previous described threshold of 264 BAU/mL [11], although including the T cell response probably allowed to obtain a better specificity.

Once below the thresholds, age and level of T cell response were the factors predictive of infection. Vaccinated individuals who got infected during the Delta-Omicron wave were younger. This could be explained by fewer booster doses in this group as it was recommended for individuals over 50 years old and probably fewer barrier gestures. We suppose that this last factor is of major importance and is the main reason why we cannot guarantee the occurrence of infection in individuals below the thresholds. Regarding the T cell response factor, the importance of the Th1 response during viral infection is well known, but in our knowledge, a level of IFNγ was never been associated to infection prediction.

The strengths of our study are (i) a large number of participants for an in-depth immune profiling study; (ii) multiple time points per participant spanning nearly 2 years of COVID-19 pandemic from the first wave of infections to the spread of omicron variant, encompassing both infection- and vaccination-related immunity against SARS-CoV-2; (iii) prospective follow-up of active individuals who could be exposed to the virus; (iv) the characterization of both humoral and cellular responses against SARS-CoV-2; (v) the establishment of thresholds for both humoral and cellular immunity that identifies bad vaccine responders in risk to infection and (vi) the heterogenicity of vaccination with or without an infection is representative of the real-life situation.

The limitations of our study include (i) important loss to follow-up between month 0 and the post month 12 questionnaire responses, which is nevertheless characteristic of the COVID-19 period with high job turnover, especially in the health care sector; (ii) our cohort was composed of working-age adults and thus cannot be extrapolated to all age categories; in addition, most of the participants were women introducing a bias in sex ratio; (iii) no neutralization data for the Omicron variant, since no commercially available surrogate virus neutralization tests were available at the time of the study and since the Omicron subvariants that emerged after are not fully recognized by the existing commercially available tests, thus limiting the usefulness of such analysis; (iv) the estimation of infection rates in our cohort was established based on a questionnaire and not on regular PCR testing and thus subject to bias such as inability to remember the exact date of a positive test, reporting COVID-19-like symptoms in the absence of COVID-19 testing, as well as inability to detect asymptomatic infections for participants who did not perform a COVID-19 test at the right moment. Nevertheless, we tried to correct for this error by systematically evaluating anti-N antibodies at each time point in order to correctly classify each participant as infected or not; (v) the variant responsible for the SARS-CoV-2 infection was often not identified in the absence of a PCR test or for the asymptomatic individuals with positive anti-N antibodies who had no knowledge of previous infection; and (vi) no severe COVID-19-related cases requiring hospitalization were reported in our study, thus precluding us from drawing conclusions on the importance of humoral and cellular biomarkers on the severity of breakthrough SARS-CoV-2 infection.

In summary, T cell response as measured by the capability of patients’ immune cells to produce Th1 cytokines such as IFNγ in response to SARS-CoV-2 antigens is more durable than humoral B cell response and seems to be variant-independent, thus providing a long-lasting and robust protection against breakthrough infection. However, considering only cellular Th1 response may not be sufficient to estimate an individual’s level of immunity, a combination of both high levels of anti-SP IgG antibodies (≥ 244 BAU/mL) and SARS-CoV-2-reactive T cells (≥ 0.285 IU/mL of IFNγ secreted) may be crucial to qualify bad responders and determine the need of a booster dose.

The thresholds for neutralizing anti-SP IgG antibodies and Th1 response to SARS-CoV-2 stimulation, taking into account the vaccination and infection history of individuals, need to be confirmed in independent studies. The threshold for sufficient cellular response should be standardized to correct for the method-specific bias as has already been done for the humoral response now reported exclusively in BAU/mL.

## Data Availability

All data are available from the corresponding author upon simple request.
